# Relaxation of the MCL after an Open-Wedge High Tibial Osteotomy results in decreasing contact pressures of the knee over time

**DOI:** 10.1007/s00167-017-4438-5

**Published:** 2017-02-15

**Authors:** N. van Egmond, G. Hannink, D. Janssen, A. C. Vrancken, N. Verdonschot, A. van Kampen

**Affiliations:** 1 Department of Orthopaedics, Radboud University Medical Centre, P.O. box 9101, 6500 HB Nijmegen, The Netherlands; 2 Orthopaedic Research Laboratory, Radboud University Medical Center, P.O. box 9101, 6500 HB Nijmegen, The Netherlands; 3 Laboratory of Biomechanical Engineering, University of Twente, P.O. box 217, 7500 AE Enschede, The Netherlands

**Keywords:** Open-wedge high tibial osteotomy, Tibiofemoral cartilage pressure, Release medial collateral ligament, Valgus laxity, Biomechanical study

## Abstract

**Purpose:**

The objective of this study was to investigate the effect of a medial open-wedge osteotomy (OWO) and the release of the superficial medial collateral ligament (MCL) on the tibiofemoral cartilage pressure, the MCL tension and the valgus laxity of the knee.

**Methods:**

Seven fresh-frozen, human cadaveric knees were used. Medial and lateral mean contact pressure (CP), peak contact pressure (peakCP), and contact area (CA) were measured using a pressure-sensitive film (I-Scan; Tekscan, Boston, MA). The MCL tension was measured using a custom-made device. These measurements were continuously recorded for 5 min after an OWO of 10°. After the osteotomy, the valgus laxity was measured with a handheld Newtonmeter. For one knee, the measurements were continued for 24 h. At the end, a complete release of the superficial MCL was performed and the measurements were repeated at 10°.

**Results:**

There was relaxation of the MCL after the osteotomy; the tension dropped in 5 min with 10.7% (mean difference 20.5 N (95% CI 16.1–24.9)), and in 24 h, the tension decreased by 24.2% (absolute difference 38.8 N) (one knee). After the osteotomy, the mean CP, peakCP and CA increased in the medial compartment (absolute difference 0.17 MPa (95% CI 0.14–0.20), 0.27 MPa (95% CI 0.24–0.30), 132.9mm^2^ (95% CI 67.7–198.2), respectively), and decreased in the lateral compartment (absolute difference 0.02 MPa (95% CI 0.03 –0.01), 0.08 MPa (95% CI 0.11 – 0.04), 47.0 mm^2^ (95% CI −105.8 to 11.8), respectively). Only after a release of the superficial MCL, the mean CP, peak CP and CA significantly decreased in the medial compartment (absolute difference 0.17, 0.27 MPa, 119.8 mm^2^, respectively), and increased in the lateral compartment (absolute difference 0.02, 0.11 MPa, 52.4 mm^2^, respectively). After the release of the superficial MCL, a mean increase of 7.9° [mean difference − 0.1° (95% CI −1.9 to 1.6)] of the valgus laxity was found.

**Conclusions:**

A release of the superficial MCL helps achieve the goal of reducing medial cartilage pressure in an OWO. There was considerable relaxation of the MCL after an OWO that resulted in a decrease of the mean CP in the medial and lateral compartments of the knee over time. However, cartilage pressure shifted from the medial to the lateral compartment only after release of the superficial MCL. The release of the superficial MCL caused a significant increase in the valgus laxity, which could influence stability after an OWO.

**Level of evidence:**

I.

## Introduction

An open-wedge osteotomy (OWO) is a successful treatment in patients with medial knee osteoarthritis (OA) and a varus leg alignment [4, 18]. An OWO unloads the medial compartment and shifts loading of the knee to the lateral compartment [1, 9, 17].

In an OWO, the medial proximal tibia must be exposed; however, the superficial medial collateral ligament (MCL) overlies this area. The superficial MCL can be left intact by elevating it sub-periostally, or it can be partially or completely released from its distal insertion [8, 11]. In literature, there is a debate as to whether or not to release the MCL when performing an OWO. Agneskircher et al. [1] concluded in their biomechanical study that if the MCL is not released after an OWO, the contact pressure in the medial compartment is even higher than in the lateral compartment. On the other hand, a release of the MCL has been shown to create a significant valgus instability [11].

It is known that ligaments show relaxation over time [5, 16], i.e. the tension in a ligament decreases over time with a constant strain. This is primarily due to maintenance of the structure in a strained condition for some finite interval of time, hence, causing some amount of plastic strain. This should not be confused with creep, which is a constant state of stress with an increasing amount of strain. The largest relaxation occurs within the first six to eight hours. After this, the effect is much smaller [5]. Theoretically, relaxation of the MCL after an OWO would result in a decrease of the cartilage pressure in the medial compartment, and the release of the superficial MCL, on that account, may not be necessary. The relaxation of the MCL and its influence on cartilage pressure has not yet been investigated.

The purpose of the present study was to investigate (1) the effect of MCL relaxation after an OWO on the contact pressure (CP), peak contact pressure (peakCP) and contact area (CA), in the medial and lateral compartments, (2) the effect of a complete release of the superficial MCL after an OWO on the CP, peakCP and CA in the medial and lateral compartments, and (3) the effect of a complete release of the superficial MCL after an OWO on the valgus laxity of the knee. It was hypothesised that (1) tension over the MCL gradually decreases, and correspondingly, the cartilage pressure in the medial compartment also decreases over time; (2) after a release of the superficial MCL, the CP, peakCP and CA in the medial compartment decreases; (3) after a release of the superficial MCL, the valgus laxity of the knee increases.

## Materials and methods

**Fig. 1:**
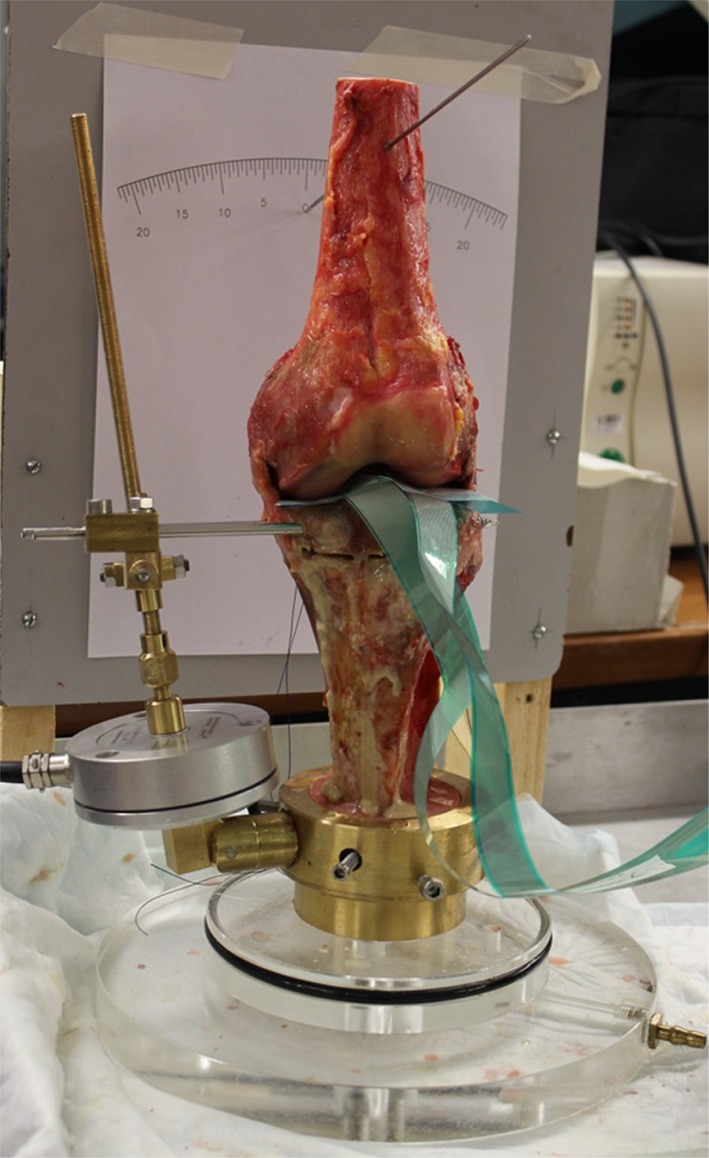
Experimental set-up with the custom-made device

Seven fresh-frozen, human cadaveric left legs were used in this study (mean age 78.9-year old (range 64–90), four men). The tibia and fibula were left as long as possible, leaving enough space for the custom-made device. The femur was cut mid-way. The cadavers were thawed over 24 h and dissected with the removal of the skin and all subcutaneous tissue. The joints were opened through a medial parapatellar approach and the quadriceps, patella, patellar tendon and anterior capsule were removed. The medial and lateral collateral ligaments (MCL and LCL), the anterior and posterior cruciate ligaments (ACL and PCL) were left intact, as was the posterior joint capsule and the popliteus tendon. The menisci were resected. The joints were visually inspected for signs of previous operations, injuries and signs of osteoarthritis. There were no signs of previous operations and injuries. Four knees had no signs of OA, one knee had mild signs of OA, and two knees had severe signs of OA, without severe osteophyte formation. None of the knees had deformities. The tibia stump was embedded in cement in a custom-made device. The femur was embedded in cement in extension, and was kept in place during cementation using a Kirschner wire (Fig. 1).

### Osteotomy

A monoplanar medial open-wedge high tibial osteotomy was carried out, without a release of the MCL. A Kirschner wire was inserted parallel to the joint line, just proximal to the tuberosity, directed to the fibular head. Along this wire the osteotomy was performed, leaving 10 millimetres of the lateral cortex intact. The osteotomy was created using a custom-made device, which was fixed to the proximal part of the osteotomy by placing screws anterior and posterior to the MCL, parallel to the osteotomy gap (Fig. 1). The gap was opened gradually until the desired osteotomy angle of 10° was reached, with one winding being equal to one millimetre. The number of millimetres c.q. windings was calculated by measuring the distance of the screw and the known desired angle (10°).

### MCL tension

The custom-made device included a tensiometer incorporated within a 5 kN force transducer (Burster 8531–5000, Burster sensors and precision measurement, Gernsbach, Germany) that was used to measure the force that was produced by the MCL (MCL tension) during the opening of the osteotomy gap. The force transducer has an accuracy of ≤±0.15% (or ≤±7.5 N). In Fig. 1, the experimental set-up is shown, with the custom-made device including the tensiometer on the left side of the cadaver. The MCL tension was measured continuously for 5 min after an osteotomy of 10° in full extension. For one knee, the measurements were continued for 24 h.

### Tibiofemoral cartilage pressure

A pressure-sensitive film [I-Scan Pressure Mapping Sensor 4000 (Tekscan, Boston, MA)] was inserted into the medial and lateral tibiofemoral joint and fixated to the posterior capsule using sutures. For protection of the sensor films, a thin piece of foil was placed over both sides of the sensor [20]. Before insertion into the joints, the sensors were preconditioned and calibrated using custom-made loading blocks in a mechanical testing system, as described in detail in other studies [19]. Care was taken to ensure that the sensors were seated on the cartilage without wrinkles. To ensure fixation, two sutures were placed through each sensor and the surrounding soft-tissue.

### Valgus laxity

The valgus laxity was measured by applying a valgus moment of 2 N m to the proximal femur, using a handheld force gauge. The angular change, [measured on a protractor placed behind the femur (Fig. 1)] caused by the valgus moment was taken as a measure for the laxity.

### Measurements

Baseline measurements of contact pressure (CP; in MPa), peak contact pressure (peakCP; in MPa) and contact area (CA; in mm^2^) of the medial and lateral compartments were continuously performed at 0° gap opening, with the knee in full extension for a period of 5 min. The MCL tension and the valgus laxity were also measured at baseline. Next, the osteotomy gap was opened to 10°, and CP, peakCP and CA were measured again, as well as the MCL tension and the valgus laxity. The measurements (MCL tension, CP, peakCP and CA) were performed continuously for 5 min. For one knee, the measurements were continued for 24 h to assess the viscoelastic effects in the longer term.

After these measurements, a complete release of the superficial MCL was performed at the level of the osteotomy. All the measurements (CP, peakCP and CA, the MCL tension and valgus laxity) were repeated, at 0° and 10°.

### Statistical analysis

Descriptive statistics were used to summarise the data. Data were given as mean and standard deviation (SD) and differences were given as mean with 95% confidence intervals (CI). Linear mixed models were used to study the effect of condition (i.e. 0° and 10° valgus opening with and without MCL release) on CP, peakCP, CA, MCL tension and valgus laxity measurements. Patient/knee was treated as random factor. Regression parameter estimates were presented with their 95% CI. *p* values <0.05 were considered statistically significant. The statistical analyses were performed using R version 3.2.2 (R Foundation for Statistical Computing, Vienna, Austria).

## Results

### Tension produced by the MCL (MCL tension)

**Fig. 2:**
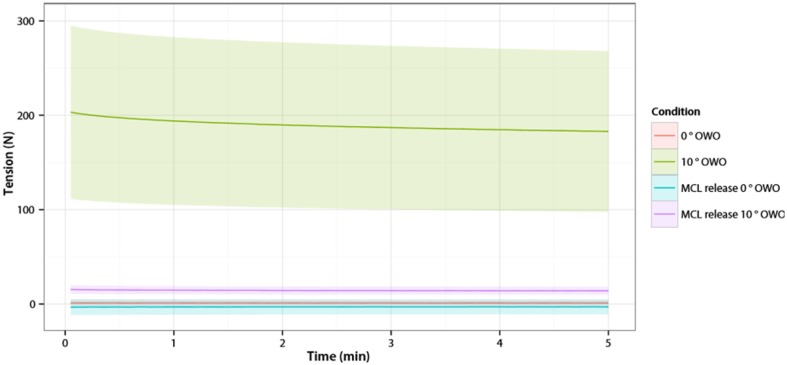
Medial Collateral Ligament tension of all knees. On the *X*-axis is the time in minutes. On the *Y*-axis is the MCL tension in Newton (mean with 95% confidence interval)

**Fig. 3:**
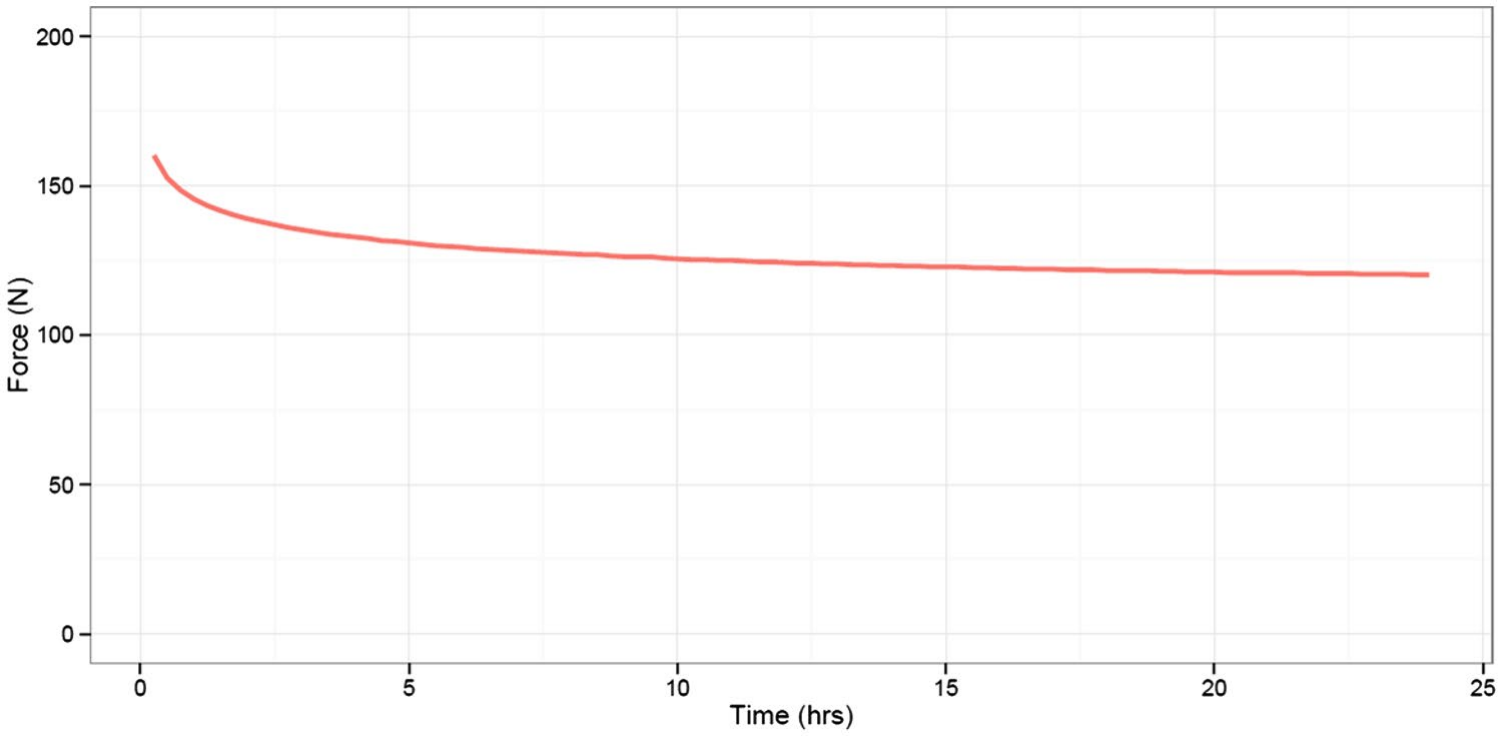
Medial Collateral Ligament tension in 24 h. On the *X*-axis is the time in hours. On the *Y*-axis is the MCL tension in Newton

At baseline, the MCL tension was 1.2 N (SD 3.8). Opening the osteotomy gap to 10° caused an average increase in the MCL tension of 203 N (95% CI 16.1–24.9) (Fig. 2). Monitoring the MCL tension after the osteotomy revealed relaxation of the MCL. In 5 min, the tension dropped with 10.7% (mean difference 20.5 N (95% CI 16.1–24.9)). In the knee that was continuously monitored for 24 h, the MCL tension decreased with 24.2% (absolute difference 38.8 N) within 24 h. In that particular knee, within the first 5 min, the tension decreased 2.1%, and between 5 min and 24 h the tension decreased an additional 22.6% (Fig. 3).

### Tibiofemoral cartilage pressures in the medial and lateral compartments

After the osteotomy, the mean CP, peakCP and CA in the medial compartment increased and in the lateral compartment decreased, compared to the situation without osteotomy (Table 1).

**Table 1:** Mean CP, peak CP, CA in the medial and lateral compartments—after 5 min compared to baseline and after MCL release

Measurements	Baseline^a^	Osteotomy 10°^a^	MCL release 10°^a^	Absolute difference osteotomy-baseline (95% CI)	*P* value	Absolute difference MCL release-osteotomy (95% CI)	*P* value
CP medial (MPa)	0.03 (0.01)	0.20 (0.07)	0.03 (0.005)	0.17 (0.14–0.20)	<0.001	−0.17 (−0.20 to −0.13)	<0.001
CP lateral (MPa)	0.03 (0.01)	0.01 (0.00)	0.04 (0.02)	−0.02 (−0.03 to −0.01)	0.001	0.02 (0.01–0.03)	<0.001
Peak CP medial (MPa)	0.10 (0.04)	0.38 (0.05)	0.11 (0.05)	0.27 (0.24–0.30)	<0.001	−0.27 (−0.30 to −0.24)	<0.001
Peak CP lateral (MPa)	0.11 (0.05)	0.03 (0.02)	0.13 (0.07)	−0.08 (−0.11 to −0.04)	<0.001	0.11 (0.07–0.14)	<0.001
CA medial (mm^2^)	193.6 (48.8)	326.5 (71.0)	203.2 (59.7)	132.9 (67.7–198.2)	<0.001	−119.8 (−185.3 to −80.6)	<0.001
CA lateral (mm^2^)	112.7 (67.1)	65.7 (100.5)	120.4 (69.6)	−47.0 (−105.8 to 11.8)	N.S	52.4 (−9.2 to 114.3)	N.S

*CI* confidence interval, *CP* contact pressure, *CA* contact area, *MCL* medial collateral ligament, *N.S*. non-significant

^a^Values given as mean (standard deviation)

After the release of the superficial MCL, the mean CP, peakCP and CA significantly decreased in the medial compartment and significantly increased in the lateral compartment compared to the osteotomy situation. In this situation, the mean CP and peak CP in the lateral compartment increased relative to the medial compartment (Table 1).

**Fig. 4:**
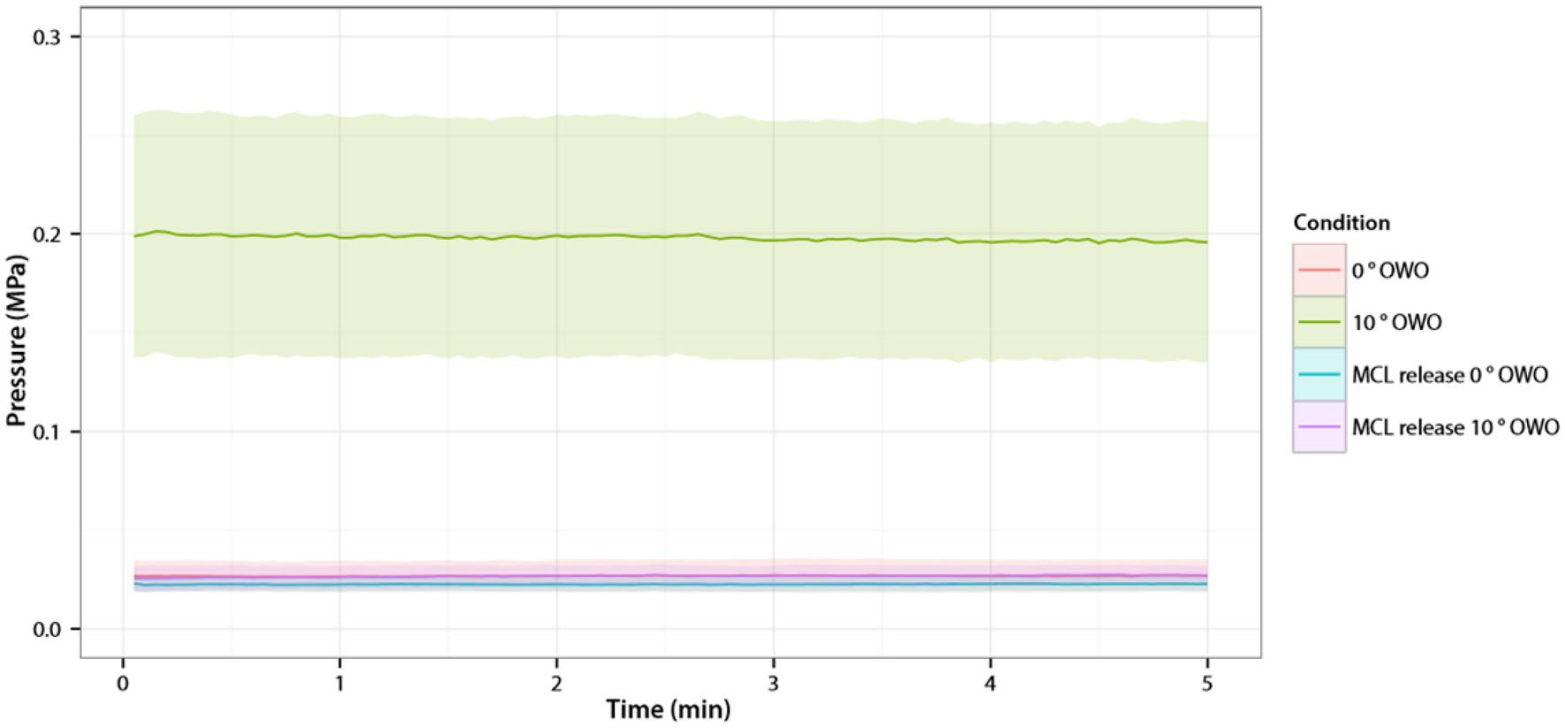
Contact Pressure medial compartment of all knees. On the *X*-axis is the time in minutes. On the *Y*-axis is the Contact Pressure in the medial compartment in MPa (mean with 95% confidence interval)

Within the first 5 min after opening the osteotomy gap to 10°, the mean CP in the medial and lateral compartments slightly decreased (1.7% (SD1.7) and 1.6% (SD3.3), respectively) (Fig. 4).

**Fig. 5:**
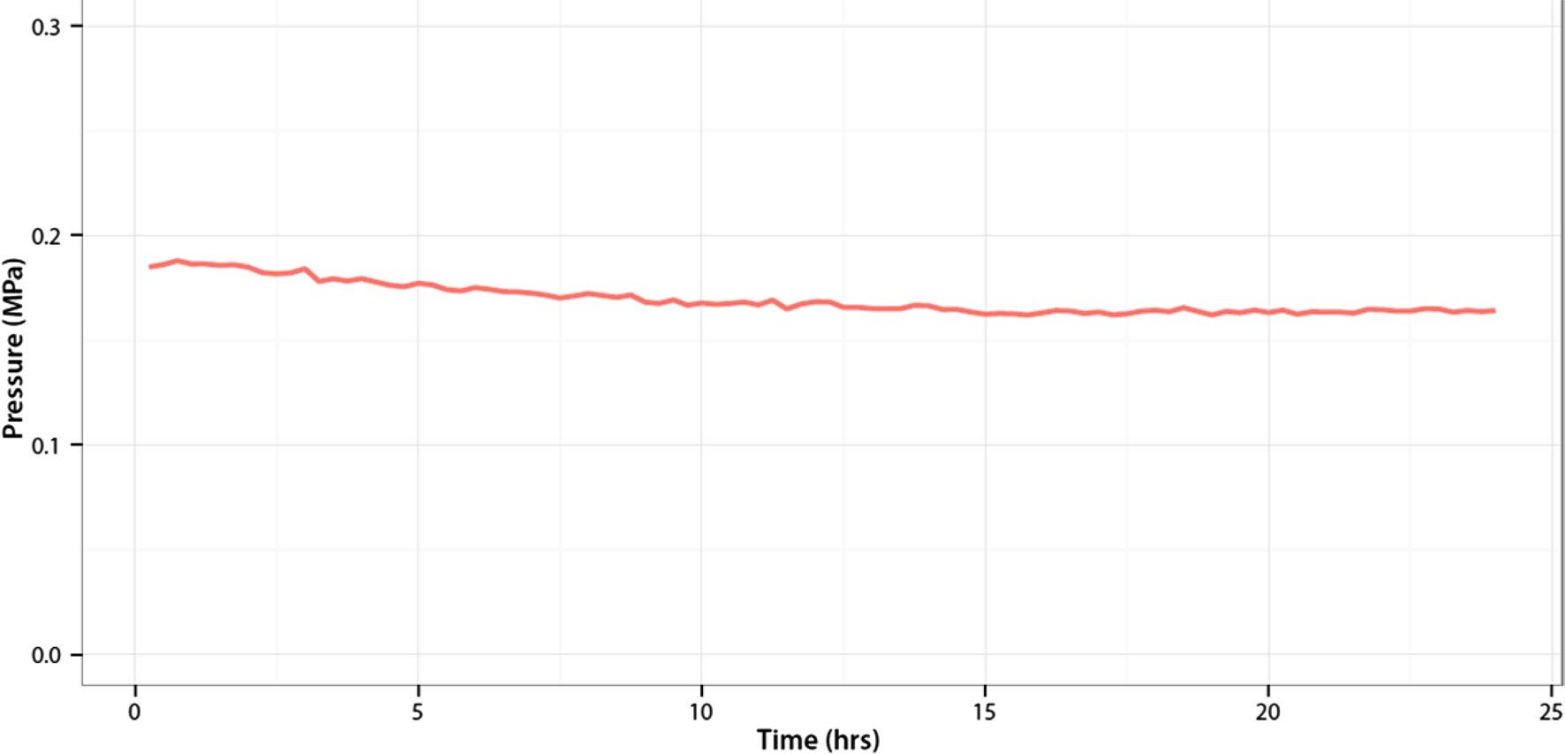
Contact Pressure medial compartment in 24 h. On the *X–*axis is the time in hours. On the *Y*-axis is the Contact Pressure in the medial compartment in MPa

In the knee that was continuously monitored for 24 h, the CP decreased within 24 h with 11.3 and 10.5% in the medial and lateral compartments, respectively (Fig. 5).

### Valgus laxity

**Fig. 6:**
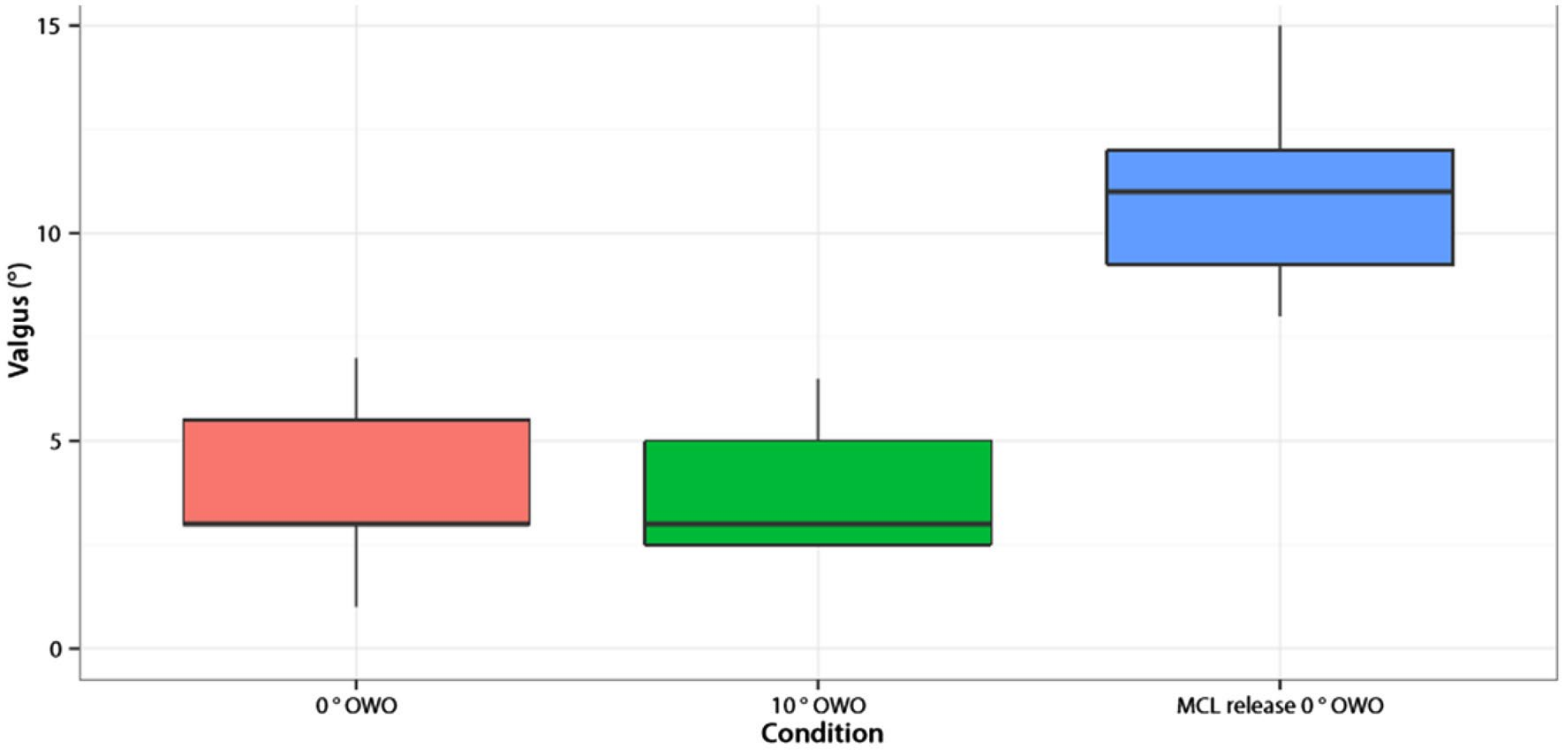
Valgus stability of all knees. On the *X*-axis are the different conditions in which the valgus stability was measured. On the *Y*-axis is the amount of valgus in degrees

Valgus laxity was unaffected by the osteotomy (mean difference − 0.1° (95% CI −1.9 to 1.6; *p* = n.s.)) alone. However, after the release of the superficial MCL, the laxity was significantly increased (mean difference 7.9° (95% CI 6.1–9.6; *p* < 0.001)) compared to the situation without OWO (Fig. 6).

## Discussion

The most important findings of this study were as follows: Firstly, there was relaxation of the MCL after an OWO resulting in a decrease of the mean CP in the medial and lateral compartments of the knee over time. Secondly, after a complete release of the superficial MCL, there was a significant decrease of the mean CP, peakCP and CA in the medial compartment and a significant increase of the mean CP, peakCP and a non-significant decrease of the CA in the lateral compartment. Thirdly, a complete release of the superficial MCL gave a significant increase in valgus laxity.

After a release of the superficial MCL, a significant decrease of the mean CP, peak CP and the CA in the medial compartment and a significant increase of the mean CP, peakCP and non-significant increase of the CA in the lateral compartment were found. These results correspond to the study of Agneskircher et al. [1]. They investigated the CP, peakCP and CA in the medial and lateral compartments with no release, a partial release and a complete release of the superficial MCL. They concluded that after a medial OWO, a complete release of the superficial MCL is required, as a shift of the cartilage pressure to the lateral compartment only occurs after this complete release. No other studies investigated the effect of a release of the superficial MCL after an OWO on the cartilage pressure. Our results confirmed the conclusions of Agneskircher et al. [1] that there is an upward mechanical lift of the medial part of the tibia plateau after an OWO without a release of the MCL, pressuring against the medial femoral condyle. They hypothesised that this leads to increased MCL tension and an associated increase of the cartilage pressure. Our results confirmed this hypothesis. We found that after an OWO of 10° there was an increase in the MCL tension of more than 200 N. The MCL consists of two components: the superficial MCL and deep MCL [12]. The tensile strength of the superficial MCL and deep MCL has been reported to be approximately 534 N and 194 N, respectively [12]. Hence, an increase of the MCL tension of more than 200 N after an osteotomy of 10° is quite a substantial amount of force.

The MCL has been described as the primary static stabiliser against valgus rotation of the knee [7, 14]. In an OWO, a release of the superficial MCL is needed for exposure and, without a release, this results in a higher cartilage pressure in the medial compartment than in the lateral compartment [1, 8]. However, a release of the superficial MCL results in a valgus instability [11, 13]. There was also a significant increase in the valgus laxity. In the present study, a complete release of the superficial MCL was performed. Pape et al. [11] investigated the presence of valgus instability after partial versus complete release of the superficial MCL by measuring the medial joint opening on stress radiographs. They concluded that the anterior fibres of the superficial MCL play a crucial role in maintaining valgus stability. Therefore, the release of the superficial MCL for an OWO should be kept to a minimum to decrease the potential of late valgus instability.

The clinical consequence of valgus laxity after a release of the superficial MCL has been recently investigated in a study by Seo et al. [15]. They explored the changes in medial laxity of the knee joint after a complete release of the superficial MCL in patients who underwent an OWO, by measuring the medial joint space opening on radiographs before, during and after surgery. They found that a complete release of the superficial MCL during OWO increases the medial joint space opening. However, the medial joint space opening decreased to the level before the release of the superficial MCL after fixing with the TomoFix plate following the opening of the osteotomy site. No significant differences were found after 3-, 6- and 12-month follow-up. Gaasbeek et al. [4] investigated the valgus stability comparing an OWO with a closed-wedge osteotomy and found that the OWO group showed a mean postoperative decrease, and not an increase, of the mean MCL laxity of 4.5° (SD 1.5) versus 5.3° (SD 1.2) in the closed-wedge osteotomy group (*p* = 0.04). However, they did not perform a release of the superficial MCL during the OWO; they shifted the superficial MCL and pes anserinus dorsally. The clinical results were equal between both groups after one-year follow-up [4]. After a follow-up of 7.8 years, there was no difference in survival rate between the OWO and closed-wedge osteotomy group [18].

A release of the superficial MCL has been shown to lead to valgus instability [11, 13]. This is in line with the findings of the present study. However, there is a discrepancy between the biomechanical and clinical findings, as a postoperative valgus instability after an OWO and release of the superficial MCL is not a common complication [6]. There are several explanations for this discrepancy. Firstly, the muscular support of the dynamic stabilisers, such as the semimembranosus tendon and the medial head of the gastrocnemius, may partially compensate for the release of the superficial MCL [13]. Secondly, there could be a re-tensioning effect of the remaining fibres of the MCL, which could restore the valgus stability [10]. Thirdly, a tendon-to-bone healing of the superficial MCL might occur during rehabilitation, thus preventing a (late) valgus instability [11].

There are several limitations to this study. Firstly, this was an experimental set-up; the measurements were not performed in vivo. Secondly, we performed our study without axial loading of the cadavers, which would have been more comparable with a clinical situation. Hence, the lateralisation of the axial force vector due to the osteotomy was not taken into account in this study. Nevertheless, even without axial loading, we found similar results to Agneskircher et al. [1], who performed a comparable study in cadaver knees, but with loading of the cadavers. Thirdly, we tested the valgus laxity only in extension. Although the MCL is the primary static stabiliser against valgus rotation of the knee, in extension the posterior medial capsule seems to be an important structure and in flexion it is the superficial MCL [3, 14]. Nevertheless, in extension, we found a significant increase in valgus laxity after release of the superficial MCL, so, in flexion, the valgus laxity is expected to be more pronounced. Fourthly, only the static stabilisers against valgus rotation were left intact, we resected all the dynamic stabilisers against valgus rotation, such as the semimembranosus tendon and the medial head of the gastrocnemius. As mentioned before, the dynamic stabilisers might partly compensate for the release of the superficial MCL. Ideally, this study would have been performed in a dynamic setting with all the stabilising structures intact. Fifthly, it is known that the tension in a ligament decreases over time with a constant strain [5, 16]. The largest relaxation is within the first six to eight hours, after that, the effect is much smaller [5]. We only investigated the relaxation and the effect on the cartilage pressure in 5 min, except for one knee, which we investigated for 24 h. The pattern of relaxation in the present study was similar to that described in the literature [5, 16]. It is expected that the relaxation of the MCL and the decrease in cartilage pressure would be higher after six to eight hours. Nevertheless, the cartilage pressure remained very high even after 24 h. Finally, we resected the menisci to fit in the pressure-sensitive film in the medial and lateral tibiofemoral compartments. This might have influenced our results, possibly an overestimation of the (peak) CP and an underestimation of the CA. In 1986, Baratz et al. [2] had already studied the effects of meniscectomy on contact areas and the stresses in the knee joints of human cadavers using pressure-sensitive film. Loss of the medial meniscus led to a decrease in contact areas of approximately 75% and an increase in the peak contact pressures of approximately 235%. Nevertheless, our results were comparable to Agneskircher et al. [1], who preserved the menisci.

The present study showed that a release of the superficial MCL is necessary for a successful OWO. Postoperatively, surgeons should consider the stability of the knee, as a release of the superficial MCL increased valgus laxity.

## Conclusion

A release of the superficial MCL helps achieve the goal of reducing medial cartilage pressure in an OWO. A considerable relaxation of the MCL after an OWO occurred, which resulted in a decrease of the mean CP in the medial and lateral compartments of the knee over time. Cartilage pressure shifted from the medial to the lateral compartment only after a release of the superficial MCL. The release of the superficial MCL caused a significant increase in valgus laxity, which could influence stability after an OWO.
